# Determinants of Weight-Related Behaviors in Male Saudi University Students: A Qualitative Approach Using Focus Group Discussions

**DOI:** 10.3390/ijerph18073697

**Published:** 2021-04-01

**Authors:** Abdulaziz Balhareth, Mohammed Jafer, Ester van der Borgh-Sleddens, Stef Kremers, Ree Meertens

**Affiliations:** 1 Department of Health Promotion, NUTRIM School of Nutrition and Translational Research in Metabolism, Maastricht University Medical Center+, 6200 MD Maastricht, The Netherlands; s.kremers@maastrichtuniversity.nl (S.K.); r.meertens@maastrichtuniversity.nl (R.M.); 2 Department of Health Education and Promotion, Faculty of Public Health and Tropical Medicine, Jazan University, Jazan 82913, Saudi Arabia; 3 Department of Preventive Dental Science, College of Dentistry, Jazan University, Jazan 82943, Saudi Arabia; dr.mjafer@gmail.com; 4 Mondriaan Mental Health Center, 6419 XZ Heerlen, The Netherlands; e.van.der.borgh-sleddens@mondriaan.eu; 5 Department of Health Promotion, CAPHRI Care and Public Health Research Institute, Maastricht University Medical Center+, 6200 MD Maastricht, The Netherlands

**Keywords:** adults, determinants, weight-related behaviors, Middle East

## Abstract

Obesity is a serious public health concern in the Gulf States. Students are exposed to many unhealthy weight-related behaviors due to college life. However, research that gives insight into regional and culture-specific aspects and determinants of weight-related behaviors in students is lacking. The purpose of this study was to explore the potential determinants of weight change, eating behaviors, physical activity, sedentary behaviors, and sleep behaviors in Saudi university students. Five semi-structured focus group discussions guided by Social Cognitive Theory were conducted, consisting of 33 male university students 20 to 22 years old. The data were transcribed, coded, and organized according to themes. The students reported weight gain due to personal, social, and environmental factors related to university lifestyle, such as unhealthy eating behaviors, low physical activity, high sedentary behaviors, and inadequate sleep. Both eating behaviors and physical activity shared similar personal aspects found in other studies, such as knowledge, stress, lack of time, and lack of motivation. However, there were some unique social and environmental factors in the region, such as the social norms, cultural aspects, weather conditions, passive transport dependency, and khat consumption, compared with studies worldwide. Such differences are key factors to developing effective interventions in the future.

## 1. Introduction

During the last four decades, overweight and obesity rates have increased in the Gulf States to an epidemic level [1]. Overweight prevalence among adults in Saudi Arabia has surged from 38.1% in 1975 to 69.7% in 2016 [1]. Over 20,000 people die every year from diseases associated with overweight and obesity in Saudi Arabia alone [2]. The Saudi government spends over EUR 4.5 billion per year fighting the burden of these diseases [3]. Other Gulf States (consisting of Bahrain, Kuwait, Oman, Qatar, and the United Arab Emirates) share similar increasing rates of overweight prevalence and its associated health care costs [1]. Overweight and obesity can be partly prevented through investing in health promotion interventions that aim to change unhealthy behaviors and promote healthy ones. Environments and communities should be structured to make the choice of healthy food and active living the most accessible, available, and affordable option. In this way, the burden of overweight and obesity can be reduced over time [4].

Recently, we performed a systematic review of the studies on correlates and interventions for overweight and obesity or weight-related behaviors among adults living in the Gulf States [5]. Correlate studies of physical activity and dietary behaviors were shown to focus mainly on sociodemographic variables. The most important and consistent correlates of overweight and obesity other than sociodemographic variables were low levels of physical activity, high sedentary behaviors, low fruit and vegetable intake, high consumption of fat and fast food, and high soft drinks consumption. In addition, some studies associated short sleep duration and sleep disturbance with overweight and obesity. The review also showed that there is a lack of research into sociocognitive, cultural, and environmental correlates of dietary behaviors and physical activity in the Gulf region [5]. Such information is important in order to understand these behaviors better.

Qualitative research in other regions of the world has indicated that eating and physical activity behaviors are determined by individual factors, social networks, and the environment [6,7,8,9,10]. However, to our knowledge, qualitative studies conducted on this topic in the Gulf States are scarce and were carried out among health professionals [11] or assessed motivators and barriers of people who had tried losing weight [12]. Some of the quantitative studies that have been carried out in the Gulf region suggest that some barriers to physical activity or a healthy diet may be more specific for the region. Barriers to being physically active in the region include hot weather and storms, lack of walking and biking trails, an urbanized environment, heavy dependency on passive transport opportunities, and norms and traditions [13,14,15,16,17]. Identified barriers that prevent a healthy diet are the consumption of traditional food that is very high in calories and the tradition of food hospitality that obliges the host to offer far too much food to guests, which they have to eat in order to avoid being impolite or disrespectful [18].

A factor that has been shown to lead to weight gain and to changes in weight-related behaviors in the Western world is the transition to college life [6,7,8]. This transition is challenging for many students due to the changes in the individual’s pattern of living as they move away from their parents’ homes and start new independent lives. Most students face difficulty regulating their eating behaviors and physical activity [6,7,8]. They often make poor decisions for basic meal planning, and their diets are low in fruit and vegetables and high in carbohydrates, fat, and sugar. An umbrella review that explored the behavioral determinants of physical activity across the life course showed that life events such as transition to university were related to low physical activity [19]. In addition, a meta-analysis suggested that more than 80% of inactive university students will continue their sedentary lifestyle later on in life [20].

An in-depth investigation is needed in the Gulf region to better understand regional and culture-specific aspects and determinants of weight-related behaviors. This understanding may feed the development of suitable and effective health promotion interventions. Therefore, the aim of this study was to explore which factors influence Saudi university students’ weight change, eating behaviors, physical activity, sedentary behaviors, and sleeping behaviors using a qualitative study design. Social Cognitive Theory was used as the guiding theory; this theory considers human behavior as the result of personal, social, and environmental factors and their interactions [21].

## 2. Materials and Methods

### 2.1. Type of Study

A qualitative study design using focus group discussions was conducted for the data collection. The study followed the “Consolidated criteria for reporting qualitative research (COREQ)” guidelines [22]. Focus groups were chosen because this method is useful to gain new perspectives and in-depth knowledge about why and how people think about a subject of interest [22]. A series of semi-structured focus group discussions was held under the direction of a trained moderator and an assistant moderator [22,23]. All focus group discussions were conducted in Arabic and led by the main researcher (AB).

### 2.2. Ethical Aspects

Ethical approval (REC39/4-S003) was obtained from the Scientific Research Ethics committee at Jazan University prior to approaching the participants.

### 2.3. Participants

Fliers, posters, word of mouth, and social media were used to recruit participants. The only inclusion criteria was that participants had to be male (as cultural norms complicate open interaction between males and females). Five focus groups were conducted with six to eight participants each. After the moderator’s presentation of the study and its objectives, the participants were asked to introduce themselves briefly, including age, study discipline, year of study, and living situation (see Table 1 for the characteristics of the participants). The participants were 33 Saudi male students aged from 20 to 22 years attending the Faculty of Public Health and Tropical Medicine in Jazan University. These participants were voluntarily recruited from four different study disciplines: Health Informatics Technology, Environmental Health, Health Education and Promotion, and Epidemiology. While the majority of students lived with their families off campus in villages nearby, some students lived in the city on campus.

### 2.4. Interview Guide

The interview guide was developed by the research team based on guidelines for focus groups [23]. There were 11 questions related to health and weight-related behaviors including eating behaviors, physical activity, sedentary behaviors, sleeping behaviors, and weight change during university time (see Table 2 for the question routing). The aim was to identify the determinants of these behaviors in order to better understand the factors that influence or shape students’ decisions to choose healthy behaviors or not. The guide was first pilot-tested in a group of eight students from the Faculty of Public Health and Tropical Medicine at Jazan University to ensure comprehension and clarity.

### 2.5. Procedure

The five focus group discussions took place in the meeting room of the Health Education and Promotion Department of the Faculty of Public Health and Tropical Medicine at Jazan University in May 2018. The time and place were chosen to suit the participants’ convenience. Each session took about 60 to 90 min and was audio recorded. Students were asked to give their consent verbally before the start of the sessions. It was the moderator’s role to make sure that the discussions were engaging, relevant, and elaborative. The assistant was responsible for observing, taking notes, and recording the discussions. Refreshments were provided during all sessions. Data saturation was reached upon the completion of the fourth focus group discussion; no new information appeared from this discussion. However, the team decided to conduct one more to be sure of not missing new perspectives. Each of the participants was given a voucher of 50 Saudi riyals (approximately EUR 12) at the end of the discussion.

### 2.6. Data Analysis

Audio recordings of each group discussion were transcribed in Arabic by the main researcher AB and double-checked by co-researcher MJ (both AB and MJ are native Arabic speakers). Quotations, sentences, or words of the transcripts were manually labelled with codes by AB to identify key concepts while preserving the context in which these concepts occurred. Then, codes were inductively clustered into themes and categories that were compared and discussed to give an overview of the main issues raised in the focus groups. Then, the analysis, theme organization, and structuring were checked by MJ., and AB and MJ discussed thoroughly whether the identified themes justified the issues raised in the focus groups. Disagreements were resolved in these discussions. By selecting, rearranging, and clustering the themes and subthemes, a reduction in the number of categories was finally attained.

## 3. Results

The results of the focus groups are presented by the themes that were discussed: weight change, eating behaviors, physical activity, sedentary behaviors, and sleeping behaviors. When relevant, the determinants influencing these behaviors are grouped into personal, social, and environmental factors. (See Table 3 for identified themes and subthemes).

### 3.1. Weight Change after Entering University

The participants indicated that many students gained weight in the transition to university, due to many factors related to university lifestyle such as unhealthy eating behaviors, low physical activity, high sedentary behaviors, and inadequate sleep. “*My cousin gained 10 kg or more since he went to university. Most students who I know have gained weight since enrolling in university*” (Fourth-year student). “*Almost all of our meals are not healthy. Food such as Kabsah and Marsah (common traditional food) unfortunately contain a lot of saturated fats*” (Second-year student). “*We know a lot of students who are lazy in general. They don’t want to be physically active*” (Fourth-year student). Moreover, students tend to eat heavy meals late at night. “*A lot of students eat late at night because they get hungry. It is very common for students to get fast food like burger, fried chicken, or pizza at midnight*” (Third-year student). Some participants stated that losing weight can also be experienced due to studying stress and not eating. “*I lost weight especially in the preparatory year because of the stressful schoolwork during that period. Some of my friends did too*” (Second-year student).

### 3.2. Eating Behaviors

Many participants stated that their eating behaviors had worsened since attending university. “I think that students do not have healthy diets. I have tried to follow a healthy diet, but there were obstacles that I faced as a student, and I think that all students face them. These obstacles can affect us, especially during exams period when unhealthy eating behaviors increase the most” (Fourth-year student). “We used to eat at specific times in high school, but now we don’t have a regular schedule anymore” (Second-year student).

#### 3.2.1. Personal Factors

##### Knowledge, Intention, and Motivation towards Diet

The students lacked knowledge about healthy eating. Some stated that individuals can eat anything they want as long as they are physically active. “*It is okay to eat unhealthy food, but you have to make up for it by being physically active*” (Fourth-year student). “*I do not have obesity or any other medical condition, so nothing holds me back from eating. Anything you eat, your body will benefit from somehow*” (Second-year student). In addition, the students lacked awareness regarding the importance of healthy food. “*Students, including myself, are not aware of what we eat. As long as it tastes good, we don’t care*” (Third-year student). Some participants assumed that most students from different colleges other than the health-related ones lack knowledge about healthy eating. “*I know more about healthy food because we learn about it in our college unlike students from different colleges where learning about healthy food is less important*” (Fourth-year student). “*Being in the field of health education has increased my knowledge unlike others and therefore influenced my eating behavior positively. Also, I was able to influence my family to make healthier choices.*” (Second-year student). “*When you know about the benefits of eating an apple, you are more likely to choose it over other unhealthy snacks*” (Fourth-year student). However, some participants believed that knowledge alone cannot change behavior. “*The behavior has to become a habit and the society as well as the environment play a role no matter how much knowledge you have*” (Fourth-year student). Most students talked about the lack of intentions to eat healthily. “*Not a lot of students intend to eat healthy. They just eat what is available*” (Third-year student). In addition, the students think that motivation can play a major role in dietary practices. The majority agreed that students lacked motivation to eat healthily. “*Nothing motivates me to eat healthy*” (Second-year student).

##### Stress and Lack of Time

University is very stressful, more so than high school as the participants stated; intense schoolwork and exams cause a lot of stress. Most students strongly believed that eating choices during such stressful periods can be affected negatively. “*Unhealthy eating behaviors tend to increase during exams period*” (Second-year student). Skipping meals was very common with a stressful schedule. One of the participants told us that he had not eaten anything yet due to an exam he had to take, and it was noon already. Some students talked about overeating. “*Many students tend to overeat when they skip a meal because they get so hungry*” (Third-year student).

Students mentioned that lack of time was a major factor influencing dietary habits. “*Healthy food preparation takes time, and being single doesn’t help because you don’t know how to cook. So, you just end up getting a burger or tortilla really fast because your time is very limited*” (Second-year student). “*If you are in a hurry, you just take anything on your way without thinking*” (Third-year student). Furthermore, living far away from the university can take a lot of time. Many students live off campus, and they drive a long distance from the university to their homes. Some students live in nearby villages and drive more than one hour to the university every day.

##### Level of Physical Activity

Some students stated that if you exercise regularly, you are most likely to choose to eat healthily. “*If you notice university students who follow healthy diets, most of them are physically active*” (Second-year student). “*What is the point of exercising and not eating healthily?*” (Second-year student).

#### 3.2.2. Social Factors

The majority of students lived with their parents or friends. They mentioned that family members and friends can impact their eating behaviors negatively. “*It is hard to follow a healthy diet when you are surrounded by family and friends who don’t because you eat with them all the time*” (Fourth-year student). They mentioned that living with their families limited their eating choices because they mostly eat what is being offered.

#### 3.2.3. Environmental Factors

##### Food Availability

The students discussed the lack of available healthy food in general. Canteens serve mainly fast food, sugar-sweetened beverages, and unhealthy snacks. Fresh fruits and vegetables, yogurt, and whole grain or wheat bread are not available. There are no opportunities to practice a healthy diet. Most students try to avoid eating on campus. “*I never eat here unless I really have to. I prefer to eat at home with my family or off campus with friends.*” (Third-year student). “*If the university provides healthy food, students will change. They will definitely eat on campus and not outside. Students will be more aware because of those changes*” (Second-year student).

The main means of transportation in the university and Jazan city is the car. Some students cannot afford to have a car or call a cab, so they end up eating on campus. “*I eat here because there are no other options. I don’t have a car to eat somewhere else*” (Second-year student). Some students indicated that there are several healthy options if you choose to eat off campus. However, the long distance along with the high prices make it more challenging for students to eat there.

##### Cultural Factors

Traditional Food

Some students talked about traditional food in general, and how attached they are to these dishes. “*Traditional food in Jazan is not the healthiest, but we definitely can’t resist having it. It tastes very good*” (Fourth-year student). “*I tried to avoid eating Marsah (type of dessert that contains a lot of fat) for two weeks, and I totally regretted it because I did not benefit from that*” (Second-year student).

Social Gathering

Another aspect the students mentioned was social gatherings and the effects on the community. In these gatherings, plenty of food is served in very large quantities in order to show generosity. Additionally, it is common for hosts to pressure their guests to overeat as a part of hospitality. One student indicated that there are some hosts who insist on offering tea (sugar is always added before serving) to a person with diabetes. Hosts sometimes offer sweets, insisting that such a small portion will not hurt. The host may feel offended if the guest does not drink or eat what is offered, leading to many complications as a result.

Cooking Skills

Another aspect students discussed was that males usually do not cook in the Saudi culture, females do. Thus, the majority of students have not learned to cook. If the students do not live with their parents, it is very common for them to eat out during that period. “*I never learned how to cook. I still live with my family so my mother or sister cooks for us all the time*” (Fourth-year student).

Social Norms

Some students talked about how they were discouraged from bringing their meals from home to the university. They said that other students might think a person is cheap if they bring their meal with them. “*Most students, including myself, do not take breakfast from home, unlike western societies where students can bring their meals to the university with them. However, it is not accepted here in our society*” (Fourth-year student).

### 3.3. Physical Activity

Participants stated that most students reduced their physical activity after starting university. “*Physical activity was mandatory in high school every morning before classes start*” (Second-year student). “*I used to play football almost every day in high school, but now I’ve stopped*” (Fourth-year student).

#### 3.3.1. Personal Factors

##### Knowledge, Enjoyment and Motivation towards Physical Activity

Students seemed to know about physical activity and its importance to health in general. However, some students had misconceptions about physical activity. They thought that exercising can make you mentally tired. “*How will the student concentrate during the lecture, he will be tired after exercising?*” (Second-year student). In addition, some students thought that exercising with the air conditioner on can be harmful. “*How do you exercise with the air conditioner is on? You definitely will get sick*” (Third-year student).

Some participants said that they do not exercise due to a lack of interest or enjoyment, as well as being tired or lazy. “*I don’t like to exercise*” (Second-year student). “*I am not motivated to exercise*” (Third-year student). “*Why should I bother to exercise?*” (Second-year student).

##### Stress and Lack of Time

Lack of time was a major issue that students talked about. They indicated that stressful schoolwork and exams are taking most of their time, making it difficult to exercise. “*We don’t have enough time to exercise; time controls us so we can’t exercise regularly*” (Second-year student). Some students added that driving the long distance to their homes takes a lot of their time. “*If you live far away, you arrive home feeling tired. You can barely find time to study and sleep*” (Second-year student).

#### 3.3.2. Social Factors

Students also talked about other external factors that consumed their time such as family responsibilities. “*I have many responsibilities besides the university like taking care of my parents’ needs, buying groceries, and taking my siblings to schools*” (Third-year student). Lack of positive social factors was an important aspect that the students discussed. “*I won’t exercise alone. I would feel bored*” (Second-year student). “*You are more likely to play football if your friends play it regularly. However, you wouldn’t play if they don’t*” (Fourth-year student). Students also mentioned that there is a lack of social activities. “*If activities are available, all students will participate*” (Third-year student).

#### 3.3.3. Environmental Factors

The students discussed the on-campus facilities and how these were being neglected. “*I haven’t seen any student using them*” (Second-year student). They mentioned that some of these facilities are not always accessible (they close early at 3:00 PM, only open two days for males and three days for females). They pointed out that there was a shortage of equipment for exercising in these facilities. Most students did not support the idea of exercising and going to the lecture afterwards. “*We came here to study and not to exercise*” (Second-year student) “*Society does not generally accept it*” (Fourth-year student). They mentioned that the facilities lacked dressing rooms, lockers, and showers. “*I don’t want to go to class after I sweat and smell bad*” (Second-year student). “*Usually if a student has some time, he will play table hockey, pool or table football. However, it is difficult to use a treadmill and avoid sweating before lectures*” (Fourth-year student). Some students talked about alternative fitness centers, but most cannot afford their pricy subscriptions. In addition, a couple of students brought up bad weather conditions. “*Heat, humid, and sometimes dust make it difficult to go outside for a walk*” (Fourth-year student). Some students discussed dependence on cars and how it restricted their physical activity. “*The environment is built for cars, nothing else*” (Second-year student).

### 3.4. Sedentary Behaviors

Students indicated that their sedentary behaviors had increased since enrolling in the university due to many factors related to the dependence on today’s technologies and rapid modernization.

#### 3.4.1. Knowledge

The majority of students were aware of sedentary behaviors and their negative effects on health. “*Students know sitting for a long time is bad for you, yet they still do it*” (Fourth-year student).

#### 3.4.2. Mandatory Sedentary Behavior

The students indicated that they had to spend a lot of time sitting for schoolwork purposes including lectures, studying, or computer use. “*We do have lectures that last up to three hours without breaks. We sit for a long time*” (Second-year student). Dependence on passive transportation, mainly cars, was indicated by students as another reason for sitting a lot. Some students mentioned sitting in the car for a long time because they live in villages nearby. “*I live in a very far place, so I drive about an hour to get here*” (Second-year student).

#### 3.4.3. Leisure Sedentary Behavior

Students talked about video games and how addictive they can be. “*I used to play football with my friends outside. Now, we just meet online and play together for hours*” (Second-year student). Many students stated that they like to watch TV. “*After a long day at the university, I just prefer to rest at home and watch a movie or two*” (Fourth-year student). “Binge watching” was common among students as well. “*My problem is that I am addicted to Netflix. I can sit all day and watch my favorite series, one episode after another*” (Third-year student). The students discussed smartphone use and its relation to sedentary behavior. “*Many students including myself spend hours on the phone using social media apps, streaming videos, and video gaming*” (Second-year student). Some students talked about khat consumption, which is part of Jazan’s culture. Khat is a type of drug that you chew. However, as it is officially illegal, it is usually consumed in small groups in a room, which means that the khat consumers just sit for many hours.

### 3.5. Sleep Behavior

Sleep duration was commonly perceived as having decreased since attending the university. Many students did not have enough sleep according to the participants, because of stress, irregular schedules, schoolwork, exams, family responsibilities, and khat consumption. Other students (especially those not living with their family anymore) mentioned that due to being new in the city and not having any social engagement after school, they had more than enough sleep.

#### 3.5.1. Knowledge

Students had adequate information about sleep and its relation to overall health. They also indicated that adequate sleep can improve productivity and increase performance. Most students suggested that an adult should sleep about 7 to 9 hours at night.

#### 3.5.2. Enjoyment

Some students mentioned that they like to stay up late to complete various tasks or to gather with family and friends for fun. “*I am a night person. I like to stay up late and get things done. I usually function better at night*” (Second-year student).

#### 3.5.3. Stress and Lack of Time

Some students revealed that they tend to postpone schoolwork until the last minute. As a result, they become stressed due to lack of time, and they do not have much sleep. “*It is impossible for me to sleep at night during finals. I prefer to stay up all night and study.*” (Fourth-year student). “*Students sleep less because they have many things to do like assignments, presentations, and projects. They can barely keep up*” (Third-year student).

#### 3.5.4. Khat Consumption

Many students reported the use of khat and how socially accepted it is in their environment. “*There is a problem in the region, which is khat consumption. It makes you sit for hours without engaging in any physical activity. Also, it keeps you awake all night, so most consumers go to university without sleep. Khat is very popular among Jazan students*” (Fourth-year student).

## 4. Discussion

The purpose of this study was to investigate which factors impact weight change, dietary habits, physical activity, sedentary behaviors, and sleep behaviors in Saudi university students. To the best of our knowledge, this is the first focus group study to explore the potential determinants of these behaviors in the region. Results could be well-structured with Social Cognitive Theory, as personal, social, and environmental factors were discussed the most.

Overall, students thought that the transition to college life was related to factors that lead to weight gain, such as unhealthy eating behaviors, low physical activity, high sedentary behaviors, and inadequate sleep. These findings are consistent with other qualitative research on determinants of weight-related behaviors among students carried out in the Western world [9].

Environmental factors within the university were mentioned as important for all weight-related behaviors but sleep. According to the students, no healthy foods were offered at the campus site. Sport facilities at the campus were only open for very limited hours, and showers, lockers, and dressing rooms were lacking. Furthermore, lectures and studying forced them to be sedentary. Studies have shown that the availability of healthy food choices and sports facilities are linked to a healthier diet and more exercise [7,9,24,25].

Dietary habits and physical activity shared similar personal factors that impeded these behaviors such as lack of knowledge and time and stress. The students showed a clear lack of knowledge, motivation, and time when it comes to eating behaviors and physical activity. Some students thought that quality and quantity of food do not matter as long as the person exercises. Additionally, misconceptions such as that exercising leads to losing one’s concentration or that you fall ill when exercising with the air conditioner on were quite common. Many students agreed that they eat unhealthy food more frequently and exercise less during stressful periods such as exam weeks. They appeared to have poor time management during such times, which led them to being stressed and consequently making unhealthy choices. Lack of motivation and lack of positive social factors (e.g., having to exercise alone) were also seen as hindering both healthy dietary habits and physical activity. Most of these findings are in line with other studies carried out in other parts of the world [6,7,8,9]. With regard to social and cultural factors, a healthy lifestyle was reported to be challenging when living with family members or friends who do not support it. Most families and friends in Jazan prefer to eat traditional food that contains a lot of calories, mainly fat and carbohydrates. Students end up having no choice but to eat with them since it is culturally offensive not to do so. There are different cultural factors that can influence eating behaviors in students, such as overeating in social gatherings, males not being trained to cook, and not bringing food from home to the university.

Previous studies found similar findings related to prolonged sitting for study purposes and screen use [8,26,27]. Compared to studies in the rest of the world, we found some different social and environmental factors that promote sedentary behavior, such as cultural aspects, weather conditions, passive transport dependency, and khat consumption. Khat is very popular among students in Jazan and is usually taken after a heavy meal in places where friends and family sit together and socialize for about 3 to 10 hours [28]. The khat leaves are chewed and stored in one side of the mouth, only the juice of the leaves is swallowed. Even though the practice of khat chewing is banned in Saudi Arabia, it is socially accepted in the region of Jazan. Khat consumption can decrease sleep because it is a stimulant and reduces physical activity; it is illegal, and therefore can only be consumed in private.

Students do not spend enough hours asleep at night due to many factors such as enjoyment, stress, lack of time, and khat consumption. Staying up late is very common among students. Nightlife encourages students to stay up late as most restaurants and cafes are open until two in the morning. In addition, it is cooler at night, which can be a motivator for students to stay up late and enjoy the relief from the day’s intense heat.

The findings of this study can support the development of health promotion interventions that target university students in regions that are comparable to the one studied here. Based on our findings, we would suggest an intervention that targets several levels at a time: the university level, the social level, and the individual level.

A university environment that promotes and facilitates healthy food choices and physical activity seems to be key. Promoting a healthy food environment with a food system that ensures the availability and affordability of diverse, balanced, and healthy diets is crucial. The university could play a major role in creating such a supportive healthy food environment that enables students to adopt and maintain healthy dietary practices easily. Furthermore, if sport facilities on campus had showers, lockers, dressing rooms, and longer opening hours, they would be used more. The university campus infrastructure could be restructured to support students to become more active unintentionally by walking and cycling to classes.

At the social level, student organizations could organize social activities that also have a physical activity and/or healthy food component, such as playing soccer or cooking courses for male students and eating a healthy meal together at a social gathering in the evening.

At the individual level, students need to adopt the idea of a healthy active lifestyle through increasing knowledge and awareness in order to avoid misconceptions, along with increasing positive social influence and support. Strategies to enhance time management could be introduced to reduce stress, as this seems to be an important factor in the unhealthy lifestyle of students.

Any intervention needs to consider these personal, social, and environmental factors in order to promote a healthy lifestyle in university students. “Health Promoting Universities” is an example of an initiative that aims to encourage universities to promote health and wellbeing through programs focusing on students, staff, and the wider community [29]. Targeting different levels and different behaviors at a time may lead students to define themselves as students of a healthy university, which may lead to social norms promoting a healthy lifestyle. To be effective, the intervention has to take cultural and regional factors into account, such as weather conditions and cultural hospitality norms.

One of the limitations of this study is the sample population, which consisted only of male students and only of university students in one region. Therefore, our results cannot be generalized to the Saudi university population. To develop an evidence-based intervention for all students, comparable studies should be conducted among female students, and in other regions. Another limitation of our study is that the data are self-reported; participants may have given social desirable answers. A third limitation is that participants volunteered to be a part of the study. Therefore, students especially interested in health issues and more knowledgeable about health-related issues may have participated. However, as the results showed, the participants had several misconceptions about health-related issues. One strength of the study is that it provided depth and details on the factors that influence weight-related behaviors in their full breadth: unhealthy eating, physical activity, sedentary behavior, and sleep quality. A future study should investigate whether the factors that seem to be weight-related behaviors in the present study are also found in a quantitative study of a larger, representative student sample.

The present study gave valuable insights into the reasons Saudi students have to perform or avoid weight-related behaviors. That part of our results was in line with those from other studies confirms that our participants wanted to discuss our topics in an open and cooperative way. Another part of the results suggested the importance of regional and culture-specific factors, adding to our knowledge, which can be used to develop regional and culture-sensitive health promotion interventions.

## 5. Conclusions

The current study concludes that university students’ weight, dietary habits, physical activity, sedentary behaviors and sleep quality behaviors can be mainly influenced by personal, social, and environmental factors. University facilities including canteens and physical activity facilities should acknowledge their role in guiding healthy behaviors and be the first to engage in creating a healthy environment for students. It is of great importance to develop evidence-based studies that target these determinants on a large scale. Evidence-based health promotion initiatives that combat these weight-related behaviors can be planned based on the findings of such studies.

## Figures and Tables

**Table 1:** Sociodemographic characteristics of the participants.

Sociodemographic Characteristics of the Participants	
**Total Participants**	Males 33
**Age (range)**	20–22
**Rural area (n)**	25
**Urban area (n)**	8
**On Campus (n)**	6
**Off Campus (n)**	27

**Table 2:** Focus group question guide.

Focus Group Questions
When do you consider a person as healthy? What is a healthy lifestyle in your opinion?
2. What do you think of health and healthy lifestyles among university students? Are students healthy in general, and do they have a healthy lifestyle? When does somebody have a healthy diet? Do fruit and vegetables have anything to do with having a healthy diet? Does snacking have anything to do with having a healthy diet, fried food, etc.? When does somebody have a healthy active lifestyle (inactivity andphysical activity)? When does somebody sleep enough?
3. How do you think eating behaviors change among students before and after enrolling in university? What do you think students’ eating behaviors in general are before enrolling in university? Where did they eat usually? What are their eating behaviors after they enrolled in university? Where do they eat? What do students in general think of the eating places at the university? Did students’ eating behavior change since enrolling in the university? Why? What factors contribute to healthy eating behaviors in university students? What factors contribute to unhealthy eating behaviors in university students? What are the challenges/barriers of healthy eating in the university? How can they be overcome?
4. How do you think physical activity changes among university students before and after enrolling in the university? What do you think students’ levels of physical activity are in general before enrolling in the university? Where did they stay physically active? For how long? What are their levels of physical activity after enrolling in the university? Where do they practice physical activity? For how long? What do students in general think of the physical activity facilities at the university? Did students’ physical activity behaviors change since enrolling in the university? Why? What factors contribute to high physical activity behaviors in university students? What factors contribute to low physical activity behaviors in university students? What are the challenges/barriers of being physically active in the university? How can they be overcome?
5. How do you think sleep habits change among students before and after enrolling in the university? What do you think students’ sleep habits are in general before enrolling in the university? Did students’ sleeping behavior change since enrolling in the university? Why? What factors contribute to sleeping enough in university students? What factors contribute to sleeping too little in university students? What are the challenges/barriers of sleeping enough in the university? How can they be overcome?
6. Has students’ body weight changed in general since enrolling at the university? Why?
7. Do students in general see overweight and obesity as a problem?
8. What factors contribute to healthy weight in university students?
9. What factors contribute to overweight and obesity in university students?
10. Which of the previously mentioned factors have the greatest influence on overweight and obesity?
11. In the future, we shall attempt to decrease overweight and obesity at Jazan University. Suppose that you were in charge, what would you change to improve healthy eating and increase physical activity and sleep duration among students at the university? What strategies do you think would work best? (changes in the environment, education or training on the risks of being overweight, education or training on how to eat healthily, education on the benefits of being physically active and sleeping, education or training on how to be physically active and how much, education on how much one should sleep)
12. Do you have anything you would like to add? Is there anything we missed?

**Table 3:** Themes and subthemes of all focus group discussions.

Themes and Subthemes of All Focus Group Discussions		
**Weight change**	Unhealthier eating behaviors after transition to university	
	Lower physical activity after transition to university	
	More sedentary behaviors after transition to university	
	More inadequate sleep after transition to university	
**Eating behaviors**	Personal factors	*Knowledge/misconceptions about importance diet for health*
		*Intention/motivation to eat healthy among students*
		*Study-related stress*
		*Lack of time (because of study or commuting)* *Not knowing how to cook*
		*Level of physical activity*
	Social factors	*Family and friend‘s influence*
		*Eating what is being offered by family or friends*
	Environmental/cultural factors	*Food availability (Lack of healthy food options on campus)*
		*Limited healthy food options off campus*
		*Cost of healthy food*
		*Distance of healthy food places*
		*Traditional food*
		*Social gathering*
		*Cooking skills*
		*Social norms*
**Physical activity**	Personal factors	*Knowledge/misconceptions about physical activity*
		*Lack of motivation/laziness*
		*Study-related stress*
		*Lack of time (because of study or commuting)*
	Social factors	*Family responsibilities*
		*Lack of possibilities to do physical activity with others*
	Environmental factors	*Limited accessibility to on-campus facilities*
		*Lack of dressing rooms, lockers, and showers in on-campus facilities*
		*Shortage of equipment in on-campus facilities*
		*Cost of alternative fitness centers*
		*Bad weather conditions*
		*Infrastructure (dependency on cars)*
**Sedentary behaviors**	Knowledge	
	Mandatory sedentary behavior	*Sitting time for schoolwork purposes*
		*Sitting time in cars for transportation*
	Leisure sedentary behavior	*Screen use, video games, social media, TV, videos*
		*Khat consumption*
**Sleeping behaviors**	Liking to stay up late	
	Studying all night or part of the night	
	Khat consumption	

## Data Availability

All relevant data are within the manuscript.
